# Economic Inequality Increases the Preference for Status Consumption

**DOI:** 10.3389/fpsyg.2021.809101

**Published:** 2022-01-07

**Authors:** Andrea Velandia-Morales, Rosa Rodríguez-Bailón, Rocío Martínez

**Affiliations:** Department of Social Psychology, Faculty of Psychology, Mind, Brain and Behavior Research Center (CIMCYC), University of Granada, Granada, Spain

**Keywords:** economic inequality, consumer behavior, status consumption, status seeking, status anxiety, materialism, indebtedness

## Abstract

Prior research has shown the relationship between objective economic inequality and searching for positional goods. It also investigated the relationship between social class and low income with conspicuous consumption. However, the causal relationship between economic inequality (the difference in wealth between individuals and groups living in a shared context and consumer behavior) has been less explored. Furthermore, there are also few studies looking for the psychological mechanisms that underlie these effects. The current research’s main goal is to analyze the consequences of perceived economic inequality (PEI) on conspicuous and status consumption and the possible psychological mechanisms that could explain its effects. Furthermore, the current research aims to examine whether there is a causal relationship between PEI and materialism preferences and attitudes toward indebtedness. This work includes two preregister experimental studies. In the Study 1 (*n* = 252), we manipulated PEI and its legitimacy through a 2 (high vs. low inequality) × 2 (Illegitimate vs. legitimate) between-participants experiment. Results showed a main effect of PEI on status consumption, status seeking, status anxiety, materialism, and attitude toward indebtedness. No interaction effect between legitimacy and inequality was found. In the Study 2 (*n* = 301), we manipulated the PEI through the Bimboola Paradigm. We replicated the effect of PEI on status consumption, status seeking, and materialism and found that status seeking mediated the relationship between PEI and status and conspicuous consumption. Economic inequality affects consumer behavior and favors consumption preferences for products that provide desirable symbolic values associated with status. These results could have important implications in the interpersonal and intergroup processes, including those related to consumption and purchase.

## Introduction

Economic inequality is evidenced by an unequal distribution of economic income and inequitable access to other resources, such as health, employment, human capital, public services, and power (Van de Werfhorst and Salverda, 2012). Economic inequality refers to the increase disparities between the incomes of the richest, middle and poorest members of society, which has grown significantly in the last decades (Stiglitz, 2012). Inequality is one factor that generates social erosion by enhancing political conflicts. For instance, economic disparities are related to high crime and mortality rates (Wilkinson and Pickett, 2009a), the increase in social distrust, and the decrease of cohesion and solidarity among groups (Sommet et al., 2018).

Although economic inequality has increased in recent years, it is also more tolerated, justified, and legitimized (Schröder, 2017; Trump, 2018). The legitimacy of inequality affects its recognition, thus the fairer the distribution of resources is perceived, more difficult it will be to detect the surrounding inequality (Rodriguez-Bailon et al., 2017). Researchers have pointed out that economic inequality (in objective terms) can be used as a cognitive anchor to estimate the ideal inequality (García-Sánchez et al., 2018b). For example, inequality is framed by focusing on the differences in resources among dis/advantaged groups. This framing could impact attitudes, perceptions, and emotions about perceived inequality as well as affect the perceived legitimacy of the wealth distribution, which in turn could impact the effects of inequality (Bruckmüller et al., 2017; García-Sánchez et al., 2018a).

In sum, inequality has important economic, political, and social effects (Wilkinson and Pickett, 2009b; Levine et al., 2010; Stiglitz, 2012; Moss et al., 2013).

However, to understand the consequences of the objective economic inequality, it is important to analyze not just objective economic inequality but perceived economic inequality (Lembregts and Pandelaere, 2014; Nishi et al., 2015; García-Sánchez et al., 2018b), for example, when analyzing its impact on decision making or consumer behavior (Walasek and Brown, 2015, 2016; Sommet et al., 2018) and the role of the psychological processes when studying these consequences (Jetten and Peters, 2019).

## Psychosocial Consequences of Economic Inequality

Research on economic inequality has grown significantly in recent years, with the purpose of identifying whether or not income inequality is the cause of many negative social consequences (Wilkinson and Pickett, 2009b). Inequality increases social uncertainty and the perception of threat in social interactions, generating negative psychological consequences (Jetten and Peters, 2019), such as status anxiety and feelings of status inferiority, which could be taken as signs of a greater need to increase individuals’ social position (De Botton, 2004). In societies with greater inequality gaps, more importance is given to income and status; there is greater social comparison, more competition for resources, as well as a greater concern for gaining status (Bowles and Park, 2005; Wilkinson and Pickett, 2009b; Corak, 2013). In this sense, differentiation based on social status generates more social distance, and encourages behaviors of status seeking, such as looking for more prestigious jobs, or purchasing social symbols that reflect status (Rege, 2008; Hopkins and Kornienko, 2009; Bertrand and Morse, 2013; Bricker et al., 2014; Walasek and Brown, 2015; Sánchez-Rodríguez et al., 2019b).

### Seeking for Social Status

Social status can be defined as the relative position that a member of a group occupies compared to others on some dimension’s society considers important: the possession of resources, physical attractiveness, wealth, or knowledge (Nelissen and Meijers, 2011). In societies with greater inequality gaps, the hierarchies are intensified and the perception of social mobility changes (Wisman, 2009). Being concerned about maintaining or increasing the social position becomes part of the personal identity and indicates social success (Rege, 2008; Wilkinson and Pickett, 2009a; Sivanathan and Pettit, 2010; Moss et al., 2013; Thal, 2020). Furthermore, in conditions of uncertainty or high social competitiveness (which are features of societies with higher levels of inequality), the status acquires a greater relative burden and triggers the motivation for acquiring and possessing the goods and resources of the reference groups (coworkers, peers, and neighbors), among others. This process of continuous comparison and concern about status can lead to high levels of stress and status anxiety (Moss et al., 2013; Paskov et al., 2013).

Status anxiety is defined as the concerns given to the relative position in the social hierarchy and is expressed by insecurity or the fear of failing to conform to society’s ideal (De Botton, 2004), and affects the status-seeking behaviors (Paskov et al., 2017). It is important to consider that status anxiety is not exclusive to the people at the bottom of the social ladder who want to ascend, but also of those who are at the top and fear falling and losing their position, thus being in constant competition for resources (Ferrer-i-Carbonell and Ramos, 2012).

Different researchers have shown that status anxiety is not derived exclusively from possessing resources, but also from the relative comparison with other reference persons and groups (Charles and Lundy, 2013). In this way, comparing the reference group members’ increased wealth with one’ own can trigger status anxiety (Ferrer-i-Carbonell and Ramos, 2012; Charles and Lundy, 2013). Therefore, living in high economic inequality conditions can increase concerns about the social position, and in turn perpetuate status anxiety (Walasek and Brown, 2019; Melita et al., 2021). Importantly, from our point of view, status anxiety should not just be considered as a consequence of economic inequality, but it could also trigger, in turn, other important psychological processes, such as consumption decisions, aimed to look for status seeking. In this line, status anxiety and status-seeking behaviors would be processes that mediate the relationship between inequality and purchasing positional goods or product brands associated with status (O’Cass and McEwen, 2004; Han et al., 2010; Walasek and Brown, 2015; Ryabov, 2016), regardless of the price of them or whether the purchase will create debt (Wisman, 2009; Nelissen and Meijers, 2011; Charles and Lundy, 2013).

Thus, status-seeking behaviors are related to improving the status and striving for a higher social position through investing in resources for returns in socioeconomic standing (Walasek and Brown, 2015). Recent research has highlighted the relationship between the purchases of positional or high-cost goods with economic inequality (Walasek and Brown, 2015, 2016; Du et al., 2021). From this perspective, positional goods are those that confer high social status to those who possess them (Walasek et al., 2018). In these studies, Walasek and Brown (2015) showed, through correlation analysis, that in the North American states where there was greater income inequality, there was also a greater online search for products related to status (jewelry, luxury clothing, and design brands). On the other hand, Du et al. (2021) found that the perception of inequality increased the pursuit of positional goods in low-status conditions (Du et al., 2021). These previous results indicate that exists a relationship between economic inequality and consumption behavior, status seeking, materialism, and spending and borrowing patterns (Bertrand and Morse, 2013; Charles and Lundy, 2013; Walasek and Brown, 2015; Ryabov, 2016). However, it is still not clear whether there is a causal relationship between these variables as well as its direction. Importantly, according to us, status anxiety and status seeking could be psychological mechanisms that contribute to explaining the relationship between economic inequality and relevant behaviors as the consumption or the purchase of status products.

### Conspicuous and Status Consumption, Materialism, and Indebtedness

Consumption decisions are a central component of everyday life and involve buying goods to meet basic needs together with gaining status (O’Cass and McEwen, 2004). The desire to gain status or social prestige from acquiring and consuming goods drives a wide range of consumer behaviors (Goldsmith et al., 1996). From the behavioral economics perspective, consumption behavior can be understood in absolute (own consumption) or relative terms (in relation to others; Alpizar et al., 2005; Frank, 2005; Carlsson et al., 2007). Some research have shown that people are concerned not just about absolute consumption but about relative one (Carlsson et al., 2007; Clark and Senik, 2010; Hillesheim and Mechtel, 2011; Clark et al., 2017) and that an important increase in utility of the consumption of products stems from the improvement of their social position (Alpizar et al., 2005). Studying relative consumption allows understanding other economic phenomena, such as saving patterns, risk behaviors, or the consumption of goods with the purpose of demonstrating wealth and success in the face of others (Becker and Murphy, 2000; Frank, 2005; Wisman, 2009). In addition, it allows an in-depth study of positional goods which are valued for the high social status conferred to the people who buy them (Frank, 2008).

In this way, purchasing decisions are linked to concerns derived from social status (Jaikumar and Sarin, 2015) and relative income (Alpizar et al., 2005). Therefore, they could be affected by economic inequality (Ordabayeva and Chandon, 2011).

Research in this field has focused on the relationship between consumer behavior and income inequality, specifically on the motivation to acquire luxury brands through, for example, Google searches and Twitter mentions (Walasek and Brown, 2015, 2016; Walasek et al., 2018) or household consumption (Perez-Truglia, 2013; Jaikumar and Sarin, 2015). These previous works found that in more unequal countries, there were more interest or mentions of luxury brands, and that income inequality increased spending on conspicuous consumption (Walasek and Brown, 2015, 2016). However, we consider it is necessary to advocate in these relationships and determine whether the levels of inequality significantly affect conspicuous and status consumption.

In the consumer psychology area, conspicuous consumption involves status or positional goods consumption. Unfortunately, conspicuous and status consumption are used interchangeably, which constitute theoretical and empirical problems (Chaudhuri and Majumdar, 2006; Amatulli et al., 2018). In the current research, we differentiate between these two constructs. Both conspicuous and status consumption refer to acquiring products that give information about the social position of the person who owns them (O’Cass and McEwen, 2004). They are both featured as acquiring goods that show wealth and the social status associated with the products becomes more important than their utility (Mazzocco et al., 2012). However, some differences distinguish them. Status consumption is focused on purchasing status symbols that signal high class and luxury, which may, in turn, increase social position (Chan et al., 2015), and is related to acquiring material goods as a sign of social success and achievement (O’Cass and McEwen, 2004). The drivers of status consumption are intrinsic, thus, an individual seeks to consume luxury products that represent their status or are in line with their lifestyle and enhance their self-esteem (Jaikumar and Sarin, 2015; Amatulli et al., 2018).

On the other hand, conspicuous consumption involves purchasing to enhance the recognition in society through goods that communicate opulence (O’Cass and McEwen, 2004; Veblen, 2005). Conspicuousness is essential if consumers want to gain approval or acceptance from their reference groups; in this sense, its drivers are more extrinsic because interpersonal influences affect them to a greater extent (Chaudhuri and Majumdar, 2006). Conspicuous consumption gives more importance to the products’ symbolic meanings (Kastanakis and Balabanis, 2014). It is described as a process covered by rational motivations in which psychological, cultural, and social aspects are intertwined (Bourdieu, 1984), thus representing and communicating values (Chaudhuri et al., 2011). In this case, the consumption level depends on not only the absolute income, but also the comparison with others (Ordabayeva and Chandon, 2011), hence consumption offers signals of uniqueness concerning others (Eckhardt and Bardhi, 2015).

To sum up, status consumption obeys internal motivations and seeks to signal an individual’s status through acquiring status-laden products and luxury brands; while conspicuous consumption is a response to a social comparison that seeks to demonstrate status by flaunting consumption and openly displaying possessions in front of others (O’Cass and McEwen, 2004).

Conspicuous and status consumption have been related to socioeconomic status and social class. Prior research has identified that people living on a low income spend a higher percentage of their earnings on products or brands to have high status or to restore feelings about their social position (Sivanathan and Pettit, 2010). Conspicuous and status consumption also been related to relative deprivation (Christen and Morgan, 2005; Eckhardt and Bardhi, 2015) *via* professing that people are unhappy to the extent that their peers have more access to consumer goods (Ordabayeva and Chandon, 2011). In this line, this research aims to go a step further and identify how the unequal distribution of income between groups affects consumer behavior. Because it may occur that in more unequal societies there is more conspicuous and status consumption.

Status goods consumption can be supported by attitudes and beliefs focused on the importance of acquiring material goods as a representation of social success (Kasser and Kanner, 2004), that is, materialism. Materialism is defined as a value from which possessions are central to achieving goals (Richins and Dawson, 1992). Materialism can affect the preferences and choices of consumption, for example, by focusing on the status that a product provides (Wang and Wallendorf, 2006). Besides, highly materialistic people assign a utilitarian function to the goods they possess, such as that they provide security, happiness, and recognition (Kim et al., 2017). High levels of materialism may have negative individual and social outcomes, such as lower personal well-being (Kasser and Kanner, 2004; Wang et al., 2020) an increase in consumerism, which may have environmental consequences (Hurst et al., 2013), or indebtedness (Garðarsdóttir and Dittmar, 2012).

In fact, borrowing is seen as a common means of having access to desired products. It is possible for an individual to spend more money than they have to access the desired goods that are considered necessary to belong to a higher status group (Denegri et al., 2012). Furthermore, having a favorable attitude toward indebtedness is related to believing in vertical mobility and low-class identity (Wisman, 2009). Additionally, the broader the income and wealth distribution gaps in society (i.e., the more inequality), the more pressure is placed on consuming goods that increase status, directly impacting savings rates (Christen and Morgan, 2005; Wisman, 2009).

In sum, as argued above and according to the social rank hypothesis proposed by Walasek and Brown (2015, 2016), economic inequality may be involved consumers preferences. More specifically, and as posited in the material rank hypothesis, economic inequality in a given context increases the awareness of the importance of material dimensions of social life as income, wealth, or having positional goods (Walasek and Brown, 2019). According to this idea, the perception of high economic inequality could generate more conspicuous and status consumption, more materialism, and indebtedness.

## Overview of the Studies

**Figure 1:**
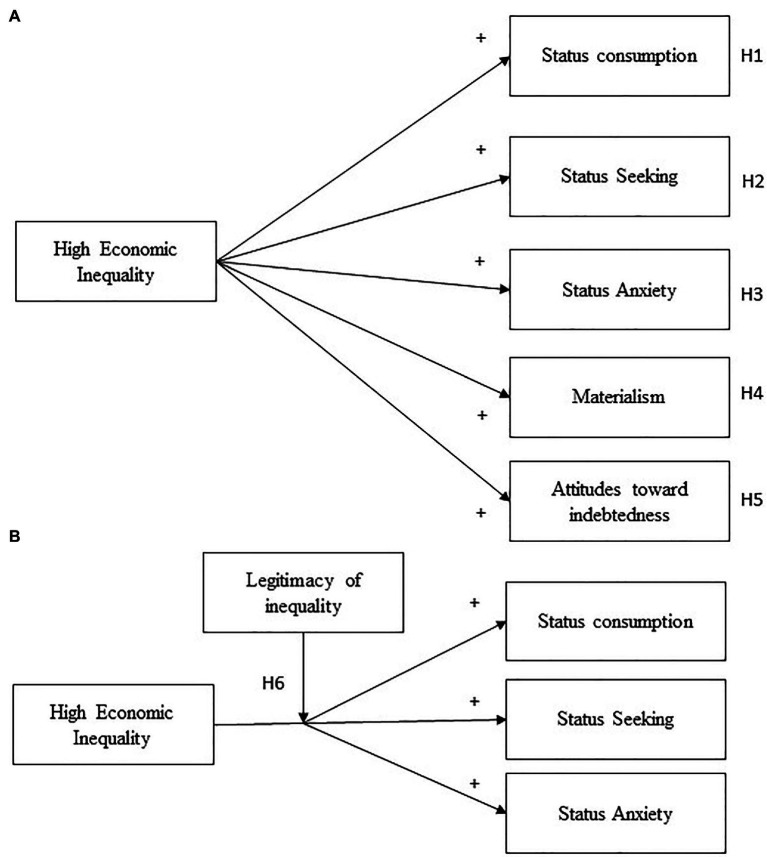
Main predictions of Study 1. Main effect of Economic Inequality on status consumption, status seeking, status anxiety, materialism, and attitudes toward indebtedness **(A)**. Interaction effect between Economic Inequality and Legitimacy of inequality on status consumption, status seeking, status anxiety **(B)**.

**Figure 2:**
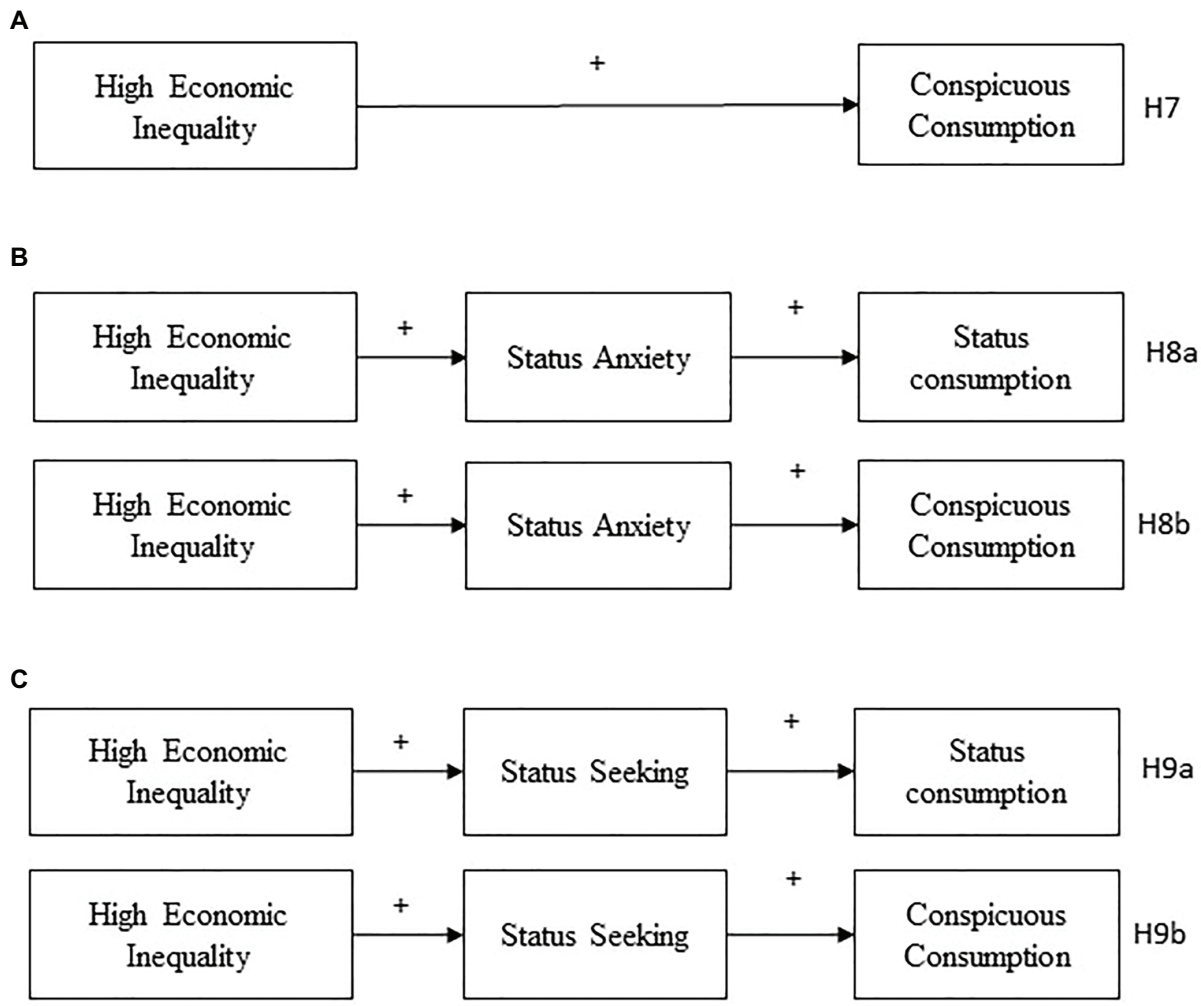
Hypotheses of Study 2. Main effect of Economic Inequality on conspicuous consumption **(A)**. Indirect effect of economic inequality on status consumption and conspicuous consumption through status anxiety **(B)**. Indirect effect of economic inequality on status consumption and conspicuous consumption through status seeking **(C)**.

This research goes beyond the relationships that exist between social class or socioeconomic status and the consumption of status-symbolizing goods which have been extensively investigated. Instead, we focus on how the perceived unequal distribution of income between groups affects consumption behavior. Thus, the current research’s main aim is to explore the effect of the perceived economic inequality on status consumption, conspicuous consumption, materialism, and attitudes toward indebtedness, and some others additional aspects related to consumer behavior, such as status anxiety and status seeking. Two experimental studies test the hypotheses. The first study analyses perceived economic inequality’s main effects on consumption behavior and its interaction with the perceived legitimacy of inequality. We predict that in the condition of high (vs. low) economic inequality there will be greater status consumption (H1), higher status seeking (H2) and status anxiety (H3), a higher level of materialism (H4), and more favorable attitudes toward indebtedness (H5). We also expect an interaction between economic inequality and its legitimacy (H6). Thus, in the high inequality legitimated condition there will be a greater preference for products related to status, more status seeking, and greater status anxiety than in the illegitimate condition. No differences in the above measures are expected in the condition of low inequality between the legitimate and illegitimate conditions (Figure 1). The second study incorporates a measure of conspicuous consumption, and we predict that in the high inequality condition there will be more conspicuous consumption (H7). Finally, Study 2 examines meditational models and predicts that status anxiety will mediate the relationship between economic inequality and status consumption (H8a), and the relationship between economic inequality and conspicuous consumption (H8b). Additionally, we expect that status seeking will mediate the relationship between economic inequality and status consumption (H9a) and between economic inequality and conspicuous consumption (H9b; Figure 2).

All hypotheses, measures, manipulations, and exclusions for the two studies were preregistered (view: https://osf.io/eg2v4/?view_only=ebcac07878ee4cb49a902e6f036c6b08).

## Study 1

This study’s purpose was to determine if there was a main effect of perceived economic inequality on status consumption, status anxiety, status seeking, materialism, and attitudes toward indebtedness and an interaction between the perception of inequality and its legitimacy. We used a 2 (economic inequality: high vs. low) × 2 (legitimacy of inequality: legitimate vs. illegitimate) between participants in a factorial design; using fictitious news about the distribution of resources in Andalusia (a region in Spain).

### Method

#### Participants

Participants volunteering for the study included 252 people from general population (151 females, *Mage* = 34.20, *SD* = 9.77). For information about education level and socioeconomic status (income and subjective status) of participants view Table 1. We conducted a sensitivity power analysis. For a mix-design ANOVA (with four groups), this sample allows us to detect an effect size as small as *f* = 0.17 (*η*^2^*_p_* = 0.02) with a power of 0.80 (and an alpha level set at 0.05).

**Table 1:** Sample’s demographic information-descriptive statistics in each experiment.

	Study 1 (*N* = 252)			Study 2 (*N* = 301)		
	*M*	*SD*	**%**	*M*	*SD*	**%**
Age	34.20	9.77		24.19	6.75	
Education Level	4.05	0.79		4.20	0.56	
**Status**						
SES (Income)	5.16	1.66		5.06	2.14	
SES-S (Subjective)	5.79	1.50		5.60	1.28	
**Gender**						
Female			61.4%			69.4%
Male			38.6%			29.3%
Other			-			1.3%

Scale Income: 1 = less than 300€; 2 = 301€ − 700€; 3 = 701€ − €1.100€; 4 = 1.101€ − 1.500€; 5 = 1.501€ − 2.000€; 6 = 2.001€ − 3.000€; 7 = 3.001€ − 4.000; 8 = 4.001€ − 5.000€; 9 = more than 5.000€ (all income per month). Education Level: 1 = primary education to 5 = university degree.

#### Procedure

We developed an online survey in the Qualtrics platform and distributed it through two different ways. We used flyers that include basic information of the study and a link to access it. We spread it among people from the general population either physically (at the center of a city in the southern of Spain) and through social networks. Participants were randomly assigned to one of the four conditions. Inequality was manipulated by presenting different fictitious news about high/low and legitimate/illegitimate levels of inequality in the distribution of resources in Andalusia (Côté et al., 2015). The legitimacy/illegitimacy of inequality was manipulated by using an adaptation of the procedure that Willis et al. (2015) used. Specifically, we presented information about the benefits/difficulties provided by inequality for the region’s development and competitiveness (for the high vs. low legitimacy conditions, respectively). All materials are available in the Methodological appendix at https://osf.io/eg2v4/?view_only=ebcac07878ee4cb49a902e6f036c6b08

Participants were asked to read news from a well-known national newspaper. Thus, depending on the experimental condition (randomized) participants were led to believe that Andalusia had a high/low level of inequality and that this high/low inequality was legitimate/illegitimate. Then, participants were asked about the study’s dependent variables. At the end of the questionnaire, they were debriefed and thanked for their participation.

#### Measures

Participants answered a questionnaire in which the following measures were included:

**Status Consumption Scale** (Griskevicius et al., 2007). Participants read the following instruction: “Imagine that you have €5,000 in your bank account and that you are considering buying a few new things. We would like to know how much money you would consider spending on each type of purchase.” Then, participants were presented with five different consumption goods: a car, a new mobile phone, a new watch, a voucher to invite a group of friends out to dinner, and a nice vacation. They indicated how much money they would spend on each of these presented five items. We used an 11-point scale for each item (α = 0.79). It is important to note that each product had a different price range. For instance, the watch and the mobile phone price ranged from € 25 to € 275; dinner with friends from € 50 to € 300; vacations from € 500 to € 3,000; and the car from € 5,000 to € 50,000. For all items, the scale had a constant increase in value between the different points of the answer scale.

**Status Anxiety**. This was measured by using the scale Day and Fiske (2016) developed and that Melita et al. (2020) adapted to Spanish. This measure is composed of five items (e.g., “I sometimes worry that I might become lower in social standing” and “I worry that my social status will not change”; 1 = strongly disagree to 5 = strongly agree; α = 0.85).

**Status Seeking**. We used the Kilsheimer Status Scale (Kilsheimer, 1993), composed of five items (e.g., “I would buy a product just because it has status” and “I would pay more for a product if it had status”; 1 = strongly disagree to 5 = strongly agree; α = 0.88).

**Materialism**. We included the Richins and Dawson Scale (Richins and Dawson, 1992) that Lado and Villanueva Orbaiz (1998) adapted to Spanish. This measure includes 12 items (e.g., “I admire people who own expensive homes, cars, and clothes” and “I do not spend money on things that are not practical”; 1 = strongly disagree to 5 = strongly agree; α = 0.83).

**Attitudes Toward Indebtedness**. This was measured using the Denegri et al. (1999) Scale. Includes 11 items divided into two orthogonal factors: indebtedness (e.g., “It is a good idea to buy something through financing plans”; 1 = strongly disagree to 5 = strongly agree; α = 0.72) and savings (e.g., “It is important to pay off debts as soon as possible”; 1 = strongly disagree to 5 = strongly agree; α = 0.61).

Finally, we also measured the subjective socioeconomic status (S-SES with the 10-steps MacArthur ladder adapted from Adler et al., 2000). We used objective SES indicators, such as monthly family income participants reported and their education level (5-point scale from “primary studies” to “university degree”). An overall measure of participants’ objective SES index was created by the sum of standardized responses of these two variables (Kraus et al., 2009).

**Demographics and Manipulation Checks**. Finally, participants provided sociodemographic information, such as the number of members in their household, occupation, age, sex, and place of residence. Manipulation checks were presented after reading the fictitious news. Furthermore, participants were asked to answer an item about perceived economic inequality, which measured their perceived degree of wealth disparity between people within a society “To what extent do you think that the distribution of the resources in Andalusia is unequal?” 1 = not at all to 7 = totally (Willis et al., 2015). The legitimacy/illegitimacy of inequality was measured by using the following item: “The inequality of resources in Andalusia has positive effects on the development of the region.” 1 = completely disagree to 7 = completely agree. At the end of the questionnaire, we also asked participants to rate the credibility of the news presented using one item “How credible were the news of the newspaper you read at the beginning of the study?” 1 = not credible to 5 = very credible.

### Results

The manipulation checks confirmed the effectiveness of the inequality manipulation, *t*(250) = −14.164, *p* ≤ 0.001, *d* = 1.77. In the high inequality condition, participants perceived more inequality (*M* = 5.60, *SD* = 1.02) than in the low inequality condition (*M* = 3.41; *SD* = 1.39). Moreover, the manipulation check’s ratings of the legitimacy of inequality manipulation also showed that it worked out, *t*(250) = −7.140, *p* ≤ 0.001, *d* = 0.87. In the legitimate condition, participants perceived more positive effects of the inequality of resources on the region’s development (*M* = 3.98; *SD* = 2.16), and in the illegitimate condition, participants perceived less positive effects (*M* = 2.25; *SD* = 1.67). Means, standard deviations, and correlations are available in Supplementary Material S1.

To test the hypotheses about the effects of the perception of economic inequality on status consumption (H1), status seeking (H2), status anxiety (H3), materialism (H4), and attitudes toward indebtedness (H5), we ran a MANOVA with inequality (high/low) as a between-participants factor. We run the Leven test to assess the homogeneity of variance of the measures used. Results showed that the Leven statistics value was significant for status consumption and status seeking measures. Thus, for these two variables, it was recommended to use a Kruskal-Wallis test in order to analyze the effect of inequality on status consumption and status seeking and (H1 and H2). In both cases, the unit of the Kruskal-Wallis test reported was the mean rank.

In this way, giving support to Hypothesis 1, we found that the Kruskal-Wallis test for status consumption, *X*^2^ = 5,194, *gl* = 1, *p* = 0.023, *η^2^_p_* = 0.019 was significant. The results showed that the high inequality condition mean rank is higher than the low inequality condition (*X* = 137.05 and *X* = 116.12, respectively). This means that in the high economic inequality condition, there was a higher status consumption than in the low inequality condition (*M* = 4.93; *SD* = 2.39, and *M* = 4.37; *SD* = 1.62, respectively). Additionally, we found statistically significant differences between conditions on status seeking, $X^{2}$ = 13,423, *gl* = 1, *p* = 0.000, *η^2^_p_* = 0.055, as well as the results showed that the high inequality condition mean rank is higher than the low inequality condition (*X* = 140.05 and *X* = 106.95, respectively). Therefore, in the high inequality condition, participants reported more status seeking than in the low inequality condition (*M* = 2.28; *SD* = 0.91, and *M* = 1.87; *SD* = 0.78, respectively), supporting H2.

On the other hand, MANOVA results showed a main effect of inequality on status anxiety *F*(1,244) = 5.190, *p* = 0.024, *η^2^_p_* = 0.021; materialism *F*(1,244) = 16.595, *p* = 0.000, *η*^2^*_p_* = 0.064, and attitudes toward indebtedness *F*(1,244) = 6.858 *p* = 0.009, *η*^2^*_p_* = 0.027; giving support to Hypotheses 3, 4, and 5. Even more interesting, and following our predictions, we found that in the high economic inequality condition, participants reported more status anxiety (*M* = 3.26; *SD* = 0.97) than the low economic inequality condition (*M* = 2.99; *SD* = 0.88; H3). In the same way, they showed themselves to be more materialist in the high economic inequality condition (*M* = 2.59; *SD* = 0.65) than in the low condition (*M* = 2.26; *SD* = 0.63; H4), and to have more favorable attitudes toward indebtedness in the high (*M* = 2.89; *SD* = 0.91) versus low economic inequality condition (*M* = 2.61; *SD* = 0.77; H5)^1^ (Table 2).

**Table 2:** Multivariate analysis for the main effect of economic inequality (high/low) and the interaction effect between economic inequality (high/low) and Legitimate (legitimacy/illegitimacy) on variables included in Study 1.

	*X* ^2^	F(1,244)	p	*η* ^2^ *_p_*
**Economic Inequality (Main effect)** *				
Status Consumption**	5.194		0.023	0.019
Status Seeking**	13.423		0.000	0.055
Status Anxiety		5.190	0.024	0.021
Materialism		16.595	0.000	0.064
Indebtedness		6.858	0.009	0.027
		*F* **(3,242)**	*p*	
**Economic Inequality × Legitimate (Interaction effect)**				
Status Consumption		0.812	0.368	
Status Seeking		0.467	0.49	
Status Anxiety		0.048	0.827	

* Economic inequality (high/low) as a between-participants factor.

** For the Status consumption and status seeking variables, the results are based on Kruskal-Wallis test.

Regarding to the interaction between the economic inequality condition and its legitimacy on dependent variables (H6), we did not find significant effects on status consumption *F*(3,242) = 0.812, *p* = 0.368; status seeking *F*(3,242) = 0.467, *p* = 0.49; or on status anxiety, *F*(3,242) = 0.048, *p* = 0.827.

**Figure 3:**
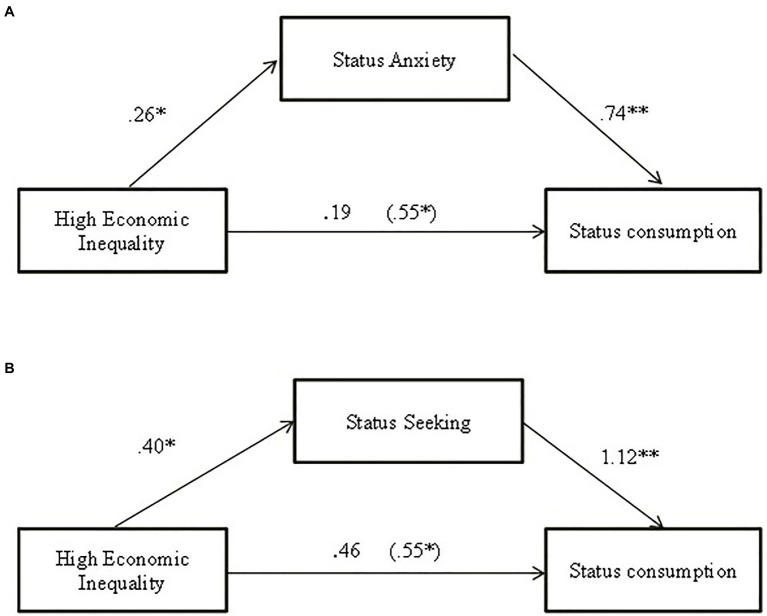
Indirect effect of economic inequality condition on status consumption through status anxiety **(A)** and status seeking **(B)** in Study 1. Coefficients are standardized: total effect in parenthesis; *p < 0.05; **p < 0.001.

Finally, in an exploratory way, we tested whether status anxiety and status seeking mediated the relationship between economic inequality and status consumption. We used the Process macro for SPSS (Model 4) using bias-corrected bootstrapping for 10,000 resamples and a 95% confidence interval (Hayes and Scharkow, 2013). First, we tested and found that status anxiety mediated the relationship between economic inequality and status consumption and, *B* = 0.1998 (0.09), [0.0410, 0.4131] (Figure 3A). Furthermore, status seeking mediated the relation between economic inequality and status consumption, *B* = 0.4615 (0.14), [0.2082, 0.7691] (Figure 3B). For a summarizes of the total, direct, and indirect effect’s, view Table 3.

**Table 3:** Total, direct, and indirect effects of the economic inequality on status consumption.

	Effect	(SE)	*t*	value of *p*	95% CI
Total effect	0.5596	0.2606	2.14	0.03	[0.46, 1.07]
**Status Anxiety as mediator**					
Direct effect	0.3595	0.2485	1.44	0.14	[−0.12, 0.84]
Indirect effect	0.1998	0.0933			[0.04, 0.41]
**Status Seeking as mediator**					
Direct effect	0.0978	0.2370	0.41	0.68	[−0.36, 0.56]
Indirect effect	0.4615	0.1422			[0.20, 0.76]

Mediation through status anxiety and status seeking in Study 1. Coefficients are non-standardized. Economic inequality (high = 1; low = 0).

### Discussion

The results of Study 1 provided evidence supporting the hypotheses that perceived economic inequality has a main effect on status consumption, status anxiety, status seeking, materialism, and attitudes toward indebtedness. These results suggest and offer empirical evidence in line with the social rank hypothesis, that economic inequality affects behavior and influences consumers’ consumption preferences (Manstead, 2018; Walasek et al., 2018).

Previous studies showed that high levels of economic inequality were related to searching for products or brands related to status (Walasek and Brown, 2015, 2016), but our results go further and provide additional findings about the causal relationship between economic inequality and status consumption, and give initial support for the concept that status anxiety and status seeking are possible consequences of economic inequality that may, in turn, explain status consumption in highly unequal contexts.

## Study 2

This study aimed to first confirm the main effect of economic inequality on status consumption, status seeking, status anxiety, and materialism obtained in Study 1 (Hypotheses 1 through 4). Additionally, in Study 2, we tested the effect of economic inequality on conspicuous consumption (H7) and the mediation role of status anxiety and status seeking on the relationship between economic inequality and status and conspicuous consumption (H8a, H8b, H9a, and H8b). All hypotheses were preregistered (see https://osf.io/247ha/?view_only=d881bd3a541148c980cf9d26f7a15be6). We used a between-participants design with two experimental conditions (high vs. low economic inequality).

### Method

#### Participants

The final sample was composed of 301 participants (206 females, *Mage* = 24.19, *SD* = 6.75), from the general population. To review information about education level and socioeconomic status (income and subjective status) of participants view Table 1. We conducted a sensitivity power analysis for a one-way ANOVA (with two groups), this sample size allows us to detect an effect size as small as *f* = 0.14 (*η*^2^*_p_* = 0.019) with a power of 0.70 (and an alpha level set at 0.05).

#### Procedure

We developed an online survey in the Qualtrics platform and distributed it through some mailing lists. The distribution message included basic information on the survey, a link to access it, and advertised that respondents to the survey would enter a draw from which they could win 50 €. Participants were randomly assigned to the experimental conditions (high vs. low economic inequality). The distribution between groups was 152 in the high economic inequality condition and 149 in the low economic inequality condition. Economic inequality was manipulated using the Bimboola Paradigm that Sánchez-Rodríguez et al. (2017) adapted. In this paradigm, participants were asked to imagine they were going to live in a fictitious society. In this society, there are three different groups according to their income (high, middle, and low). In the high inequality condition, the income gap between groups is very large: The group of high income earns 13,500 Bimboolean coins per month (BC/m), whereas the group of low-income earns 500 BC/m. Conversely, in the low inequality condition, the gap between the high/low incomes is smaller: The group of high income earns 8,000 BC/m, and the group of low-income earns 6,000 BC/m. In both conditions, all participants were assigned to the middle-income group, which earned 7,000 BC/m. To reinforce the manipulation, participants were shown houses, cars, and vacations of the different income groups and then were asked to choose a house, a car, and a vacation plan according to their income (they could choose just those in the middle- or low-income group) to start a new life in Bimboola. Finally, participants responded to a questionnaire that included the dependent variables. At the end of the questionnaire, they were debriefed and thanked for their participation.

#### Measures

We used the same scales to measure the same variables as the ones included in Study 1 (status consumption, α = 0.66; materialism, α = 0.82, and status anxiety, α = 0.79). However, in Study 2, we measured status seeking using the Fundamental Social Motives Inventory (Neel et al., 2016) to have a measure of social status in general and not exclusively associated with consumption. This measure included 6 items (e.g., “It’s important to me that others respect my rank or position” and “I do not like being at the bottom of a hierarchy”; 1 = strongly disagree to 7 = strongly agree; α = 0.79).

We added another measure of status consumption based on the factors O’Cass and McEwen (2004) proposed. In contrast to Griskevicius et al. (2007) scale used in Study 1, this measure included a cognitive component of status consumption. This scale had 5 items (e.g., “The products I buy must be a symbol of the prestige I have in Bimboola” and “The products I buy reflect my achievements at Bimboola”; 1 = strongly disagree to 5 = strongly agree; α = 0.89). We used the Social Consumption Motivation Scale (Moschis, 1981) to measure conspicuous consumption. This scale has two orthogonal factors (conspicuous and objective consumption). Bearing in mind this research’s goals, we just used the conspicuous dimension. This dimension had four items: “Before purchasing a product at the mall, it is important to know…what friends think of different brands or product; what kind of people buy certain brands or products; what others think of people who use certain brands or products; and what brands/products to buy to make good impressions on others”; 1 = strongly disagree to 5 = strongly agree; α = 0.793.

Subjective and objective socioeconomic status was measured as in Study 1.

**Demographics and manipulations checks**. Participants provided sociodemographic information, such as the number of members in their household, occupation, age, and sex. Manipulation checks were presented after the description of Bimboola society. We asked participants: “To what extent is Bimboola’s economic distribution unequal/equal?” (reversed item: 1 = somewhat unequal/equal to 9 = very unequal/equal; *r* = 0.84; Eisinga et al., 2013). As an additional manipulation check, we asked the participants to which group they had been assigned.

### Results

The manipulation checks confirmed that the Bimboola manipulation was effective. As we expected, in the high inequality condition, participants perceived more inequality than in the low inequality condition *t*(299) = −19.837, *p* ≤ 0.000, *d* = 2.25 (*M* = 8.01; *SD* = 1.39; *M* = 4.51; *SD* = 1.66, respectively). Means, standard deviations, and correlations are available in Supplementary Material S2.

To test the preregistered hypotheses, we ran a MANOVA to replicate the main effects found in Study 1 (Hypotheses 1 to 4) and Hypothesis 7. Regarding the status consumption, by using the new scale added in this study (based on the factors O’Cass and McEwen), we found a main effect of economic inequality, *F*(1,299) = 4.871, *p* = 0.028, *η*^2^*_p_* = 0.02. Thus, in the high economic inequality condition (vs. low), participants reported more status consumption in Bimboola, (*M* = 1.99, *SD* = 0.76; *M* = 1.79, *SD* = 0.78, for high versus low economic inequality conditions, respectively), supporting Hypothesis 1. However, by using Griskevicius et al.’s (2007) scale, we did not find a significant main effect [*F*(1,299) = 2.323, *p* = 0.129]. In relation to Hypothesis 2, results showed a main effect of economic inequality on status seeking *F*(1,299) = 7.003, *p* = 0.009, *η*^2^*_p_* = 0.023. Specifically, we found that in the high economic inequality condition (vs. low) participants reported more status seeking (*M* = 4.12; *SD* = 1.16; *M* = 3.76; *SD* = 1.21, for high vs. low economic inequality conditions, respectively), giving support to Hypotheses 2. As regards status anxiety, we did not find a main effect of the economic inequality *F*(1,299) = 2.034, *p* = 0.155 contrary to Hypotheses 3. About materialism, we found a main effect of economic inequality *F*(1,299) = 5.326, *p* = 0.022, *η*^2^*_p_* = 0.02, this means that in the high economic inequality condition (vs. low) participants reported more materialism (*M* = 2.44; *SD* = 0.58; *M* = 2.28; *SD* = 0.65, for high vs. low economic inequality conditions, respectively), supporting Hypotheses 4.^2^

Finally, we did not find a main effect on conspicuous consumption, *F*(1,299) = 1.275, *p* = 0.260, contrary to Hypotheses 7 (Table 4).

**Table 4:** Multivariate analysis for the main effect of economic inequality (high/low) on variables included in Study 2.

	*F*(1,299)	*p*	*η* ^2^ *_p_*
**Economic Inequality (Main effect)**			
Status Consumption Griskevicius, et al. Scale	2.323	0.129	
Status Consumption Based on O’Cass and McEwen	4.871	0.028	0.02
Status Seeking	7.003	0.009	0.023
Status Anxiety	2.034	0.155	
Materialism	5.326	0.022	0.02
Conspicuous Consumption	1.275	0.260	

Economic inequality (high/low) as a between-participants factor.

**Figure 4:**
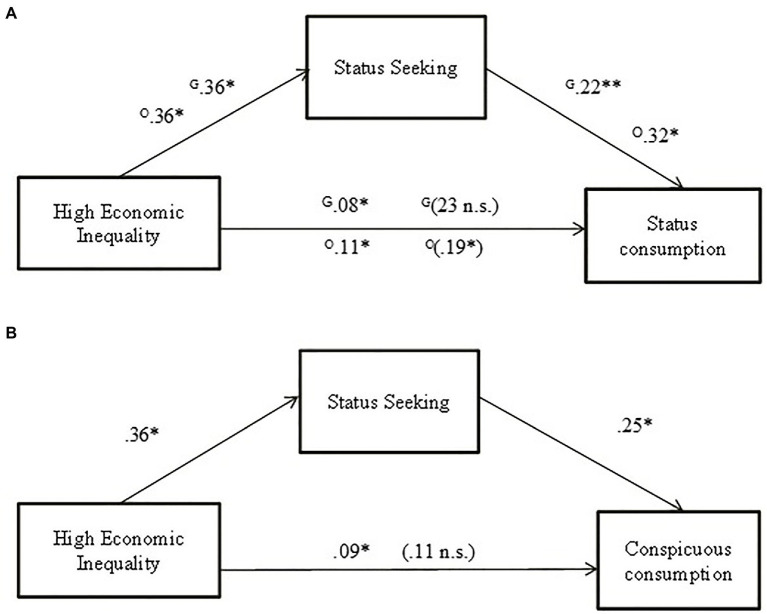
Indirect effect of perceived economic inequality condition on status consumption **(A)** and conspicuous consumption **(B)** through status seeking in Study 2. **(A)** Shows the coefficients for the two measures of status consumption used. Coefficients (standardized) of the mediation with Griskevicius et al. (2007) status consumption scale are identified with a G, and coefficients (standardized) of the mediation with status consumption scale based on the factors O’Cass and McEwen (2004) are identified with a O; total effect in parenthesis; *p < 0.05; **p < 0.001.

To test whether status anxiety and status seeking mediated the relationship between economic inequality and status and conspicuous consumption, we used the Process macro for SPSS (Model 4) using bias-corrected bootstrapping for 10,000 resamples and a 95% confidence interval (Hayes and Scharkow, 2013). First, we tested if status anxiety mediated the relationship between economic inequality and both measures of status consumption (H8a). In the first mediation, we used Griskevicius et al.’s (2007) status consumption scale as a criterion variable and did not find the predicted indirect effect, B = 0.0066 (0.01), [− 0.0157, 0.0688]. Secondly, we used the new scale of status consumption included in this study, and we did not find an indirect effect, B = 0.0355 (0.02), [−0.0094, 0.0901]. Then, we tested if status anxiety mediated the relationship between economic inequality and conspicuous consumption, but again, the predicted indirect effect did not emerge, B = 0.0357 (0.02), [−0.0096, 0.0981]. Those results did not support Hypotheses 8a and 8b. Afterward, we tested whether status seeking was a significant mediator for the relationship between economic inequality and the two measures of status consumption used (H9a). In this case, as predicted, we found an indirect effect, with the Griskevicius et al. (2007) status consumption scale, *B* = 0.0827 (0.04), [0.0203, 0.1934] also with the status consumption scale based on the factors O’Cass and McEwen (2004) provided, *B* = 0.1164 (0.04), [0.0330, 0.2068] (Figure 4A). In the same line, status seeking mediated the relationship perceived economic inequality and conspicuous consumption (H9b), *B* = 0.0914 (0.03), [0.0273, 0.1724] (Figure 4B). In sum, we found support to the Hypothesis 9a and 9b. For a summarizes of the total, direct, and indirect effect’s, view Table 5.

**Table 5:** Total, direct, and indirect effects of the economic inequality on status consumption and conspicuous consumption.

	Status Consumption Griskevicius, et al. Scale				Status Consumption Based on O’Cass and McEwen				Conspicuous Consumption			
	Effect (SE)	*t*	value of *p*	95% CI	Effect (SE)	*t*	value of *p*	95% CI	Effect (SE)	*t*	value of *p*	95% CI
**Status Anxiety as mediator**												
Total effect	0.23 (0.15)	1.52	0.12	[−0.06, 0.54]	0.19 (0.08)	2.20	0.02	[0.02, 0.37]	00.11 (0.09)	1.12	0.25	[−0.08, 0.30]
Direct effect	0.23 (0.15)	1.47	0.14	[−0.07, 0.53]	0.16 (0.08)	1.87	0.06	[−0.00, 0.33]	0.07 (0.09)	1.87	0.06	[−0.00, 0.33]
Indirect effect	0.00 (0.01)			[−0.01, 0.06]	0.03 (0.02)			[−0.00, 0.09]	0.03 (0.02)			[−0.00, 0.09]
**Status Seeking as mediator**												
Total effect	0.23 (0.15)	0.52	0.12	[−0.06, 0.54]	0.19 (0.08)	2.20	0.02	[0.02, 0.37]	0.11 (0.09)	1.12	0.25	[−0.08, 0.30]
Direct effect	0.15 (0.15)	0.99	0.31	[−0.14, 0.45]	0.08 (0.07)	1.02	0.30	[−0.07, 0.23]	0.02 (0.09)	0.21	0.82	[−0.16, 0.20]
Indirect effect	0.08 (0.04)			[0.02, 0.19]	0.11 (0.04)			[0.03, 0.20]	0.09 (0.03)			[0.02, 0.17]

Mediation through status anxiety and status seeking in Study 2. Coefficients are non-standardized. Economic inequality (high = 1; low = 0).

### Discussion

Study 2 replicated the findings of Study 1 regarding the effects of perceived economic inequality on status consumption, materialism, and status seeking. Furthermore, it provides evidence of a psychological mechanism that could explain the relationship between perceived economic inequality and consumption, whether conspicuous or status consumption. Importantly, in this sense, Study 2 allowed us to identify that status seeking mediated the relationship between perceived economic inequality and (a) conspicuous consumption and (b) status consumption.

## General Discussion

This research aimed to test in an experimental setting the causal relations between perceived economic inequality and consumer behavior, specifically the effects of perceived economic disparities on status and conspicuous consumption, status anxiety, status seeking, materialism, and attitudes toward indebtedness. The findings provided empirical evidence about how the perception of economic inequality affects these variables and offers relevant information about status seeking as a sociopsychological mechanism involved in this relationship. This is the main contribution to the current literature in the field, which thus far has shown correlations between economic inequality and online searches of status goods and luxury brands (Walasek and Brown, 2015, 2016). These results also provide evidence that goes beyond the effects of absolute income on consumer behavior on the preference of status products, and conspicuous consumption (Wang and Wallendorf, 2006; Sivanathan and Pettit, 2010).

Study 1 revealed that the perception of economic inequality affects status consumption, status seeking, status anxiety, materialism, and attitudes toward indebtedness. Study 2 replicated the causal relation between perceived economic inequality and status consumption, status seeking, and materialism. Moreover, Study 1 supported the status anxiety hypothesis that proposed economic inequality conditions would the concerns about social status (Wilkinson and Pickett, 2009b), though the relative ranked position derived from the social comparison (Walasek and Brown, 2019). In turn, this leads people who live in higher economic inequality conditions to engage in more status-seeking behaviors (Wang et al., 2019) to maintain or climb the social ladder (Buttrick and Oishi, 2017).

These results also show that although consumer behavior is generally explained by psychological and social factors, it is important to consider structural variables, such as economic inequality that can affect individuals’ consumer behaviors. Thus, it points out the importance of structural factors, which can affect consumers’ opinions and intentions supporting the approach of the social rank hypothesis (Walasek et al., 2018; Dubois et al., 2021).

Furthermore, our findings offer empirical evidence for what Walasek and Brown (2019) called the Material Rank Hypothesis. We found that inequality makes people more materialistic perhaps because material goods act as “status symbols” that signals their own social rank to others (Wang and Wallendorf, 2006; Kraus and Keltner, 2009; Goldsmith and Clark, 2012; Kraus et al., 2017). In the same way, attitudes toward indebtedness are more positive and favor purchasing material goods (Christen and Morgan, 2005) that are used to signal desired status (Kraus et al., 2017). These results can be related to previous findings about the effect of economic inequality on the normative climate. Several findings suggest that societies with high economic inequality are more competitive and increase the importance given to money and wealth (Jin et al., 2011; Sánchez-Rodríguez et al., 2019a,b). Consequently, in these societies, the concern for wealth and social status increases, and social comparison based on material goods is encouraged, thus it would not be surprising that the preference and purchase of positional goods were more frequent in these contexts.

According to Jetten and Peters (2019), when researching the role of psychological processes, it can be helpful to understand how to relate the inequality between individuals and groups, for example through social comparison. In this sense, this research provides evidence about status seeking as a psychological mechanism that can explain the relationship between perceived economic inequality and consumption. That is, high economic inequality triggers status-seeking behaviors, and these in turn generate more status and conspicuous consumption. These findings can be interpreted under the compensatory consumption theory, which argues that consumption can serve to compensate for social status that has not been achieved from other sources, for example, employment, income, or social prestige (Grønmo, 1988). According to this theory, status and conspicuous consumption are triggered by the desire to have as much as the group’s individuals compare to or to use the products they use, thus achieving a higher social position (O’Cass and McEwen, 2004; Chaudhuri et al., 2011). Thus, our results point out the importance of status in unequal perceived contexts, which could explain why status-seeking behaviors evidenced by consumption may have a compensatory function that satisfies psychological needs, such as achieving higher social standing (Mazzocco et al., 2012). However, future research may include the manipulation of status seeking since that would provide a stronger claim for the causal relationship between this variable as mediator and status or conspicuous consumption as dependent variable.

In both studies we found that when economic inequality was high, individuals were more motivated by status consumption, but not by conspicuous consumption. These results possibly indicate a difference between these two constructs in line with what O’Cass and McEwen (2004) suggested. On the one hand, as stated above, status consumption is more related to purchasing products that signal status, symbols that increase social position, or that reflect prestige, social success, or achievements (Chan et al., 2015). On the other hand, conspicuous consumption aims to communicate opulence (O’Cass and McEwen, 2004; Veblen, 2005), gain recognition, approval, or acceptance from the reference groups (Mazzocco et al., 2012; Kastanakis and Balabanis, 2014). Regarding conspicuous consumption, it is important to know which brands or products make a good impression on others or what kind of people buy preferred brands or products (Moschis, 1981). Thus, it is possible that in the absence of information on social dynamics and reference groups in Bimboola, the effect on conspicuous consumption did not emerge.

A limitation of our research is related to the results found about status anxiety. In Study 1 we observed that in conditions of high perceived economic inequality, participants reported higher status anxiety and that it mediated the relationship between inequality and both status and conspicuous consumption. However, the results of Study 2 did not confirm either the main effect of inequality on status anxiety or its mediating role on status or conspicuous consumption. These differences between both studies’ may be due to the procedure used in Study 2. In this study we asked participants to imagine themselves in a fictitious society (Bimboola), which they did not know. The information displayed about Bimboola referred to three social groups divided by income (high, medium, and low) and associated with specific products (houses, cars, and vacations). However, we did not offer information about employment, friends, family, or possible social interactions, thus it is possible that the manipulation was not strong enough to affect the anxiety about losing social status. Bearing this in mind, further research should investigate these results’ possible explanation while maintaining ecological validity.

Another limitation of the current studies regards the role of the legitimacy of inequality. We expected there would be an interaction effect between legitimacy and perceived economic inequality, but this effect was not found. This may be due to the way in which we manipulated legitimacy. In the current research, the information presented referred to the benefits/difficulties that inequality provided for the development and competitiveness of one region, and it is possible that this argument does not adequately represent what the legitimization of economic inequality means. Another possible reason is that Study 1 may be under-powered as interaction effects need bigger samples (Giner-Sorolla, 2018; Lakens and Caldwell, 2019). In this respect, it will be interesting to carry future studies with different legitimacy manipulations using bigger simple sizes.

Despite the limitations, this research contributes to understanding the psychosocial consequences of economic inequality, specifically in the field of consumer psychology. In this way, we were able to delve into how economic inequality shapes consumer preferences and how it can affect consumer behavior. Thus, economic inequality promotes conspicuous and status consumption as an adequate response to adapt to highly unequal environments in which social comparison and competition are highly relevant, which in turn could keep a high perception of inequality (Sánchez-Rodríguez et al., 2019a,b). For this reason, we consider it important to design intervention programs to raise awareness of the negative effects of economic inequality, as well as to create messages aimed at reducing the importance of the acquisition of material goods as a continuous form of social competence.

## Conclusion

In conclusion, our research’s results revealed that economic inequality, that is, the difference between who have more and less wealth in a given context, influences consumption preferences. Economic inequality favors consumption preferences for products that provide desirable symbolic values associated with status. Additionally, the current findings support the role of status seeking to explain the link between economic inequality and status or conspicuous consumption. Our findings add to the literature about the effects of economic inequality on consumer behavior, providing new lines of research, such as on the role of status seeking or the compensatory use of consumption in conditions of high inequality. Thus, the current research’s results contribute to understanding the impact of inequality on all types of interpersonal and intergroup processes, including those related to consumption and purchase.

## Data Availability Statement

The datasets presented in this study can be found in online repositories. The names of the repository/repositories and accession number(s) can be found at: https://osf.io/eg2v4/?view_only=ebcac07878ee4cb49a902e6f036c6b08.

## Ethics Statement

The studies involving human participants were reviewed and approved by Human Research Ethics Committee of the UGR. The patients/participants provided their written informed consent to participate in this study.

## Author Contributions

AV-M, RR-B, and RM conceived and designed the studies. AV-M carried out the studies, collected and analyzed the data, and wrote the manuscript with support of RR-B and RM. All authors contributed to the article and approved the submitted version.

## Funding

This research was supported by the Spanish Ministry of Science and Innovation under Grant (PID2019-105643GB-I00/SRA/10.13039/501100011033) and Grant (PCI2020-112285); and Junta of Andalucía under Grant (B-SEJ-128-UGR18).

## Conflict of Interest

The authors declare that the research was conducted in the absence of any commercial or financial relationships that could be construed as a potential conflict of interest.

## Publisher’s Note

All claims expressed in this article are solely those of the authors and do not necessarily represent those of their affiliated organizations, or those of the publisher, the editors and the reviewers. Any product that may be evaluated in this article, or claim that may be made by its manufacturer, is not guaranteed or endorsed by the publisher.

## Footnotes

^1^MANOVA analysis was repeated controlling for Objective SES. The results allow us to argue that the effect of PEI on status consumption (*p* = 0.006), status seeking (*p* = 0.000), status anxiety (*p* = 0.021), materialism *(p* = 0.000), and attitudes toward indebtedness *(p* = 0.002) remained when controlling for SES.

^2^MANOVA analysis was repeated controlling for Objective SES. The results allow us to argue that the effect of PEI on status consumption (*p* = 0.020), status seeking (*p* = 0.006), and materialism *(p* = 0.024) remained when controlling for SES.
