# Supplementary material for: Economic Inequality Increases the Preference for Status Consumption
Source: Front Psychol. 2022 Jan 7;12:809101. doi: 10.3389/fpsyg.2021.809101 (PMC8777289; doi:10.3389/fpsyg.2021.809101)
Supplement: Supplementary file 1 [file Data_Sheet_1.docx]

Supplementary Material

# Supplementary analyses in Study 1 and Study 2

# Supplementary analyses in Study 1

## Descriptive statistics and Correlations in Study 1

Table S1.
*Descriptive statistics and Correlations between variables in Study 1*

|  | M | SD | *1* | *2* | *3* | *4* | *5* | *6* | 7 |
| --- | --- | --- | --- | --- | --- | --- | --- | --- | --- |
| *1. Status Consumption* | 4.62 | 2.05 | - | 0.360^***^ | 0.485^***^ | 0.458^***^ | 0.429^***^ | 0.206^**^ | 0.340^***^ |
| *2. Status Anxiety* | 3.13 | 0.93 |  | - | 0.444^***^ | 0.573^***^ | 0.297^***^ | -0.129 | -0.005 |
| *3. Status Seeking* | 2.07 | 0.87 |  |  | - | 0.744^***^ | 0.454^***^ | 0.077 | 0.144^*^ |
| *4. Materialism* | 2.43 | 0.66 |  |  |  | - | 0.484^***^ | -0.002 | 0.091 |
| *5. Indebtedness* | 2.74 | 0.85 |  |  |  |  | - | 0.032 | 0.089 |
| *6. SES (Objective)* | 0.0029 | 1.59 |  |  |  |  |  | - | 0.415^***^ |
| *7. S_SES (Subjective)* | 5.79 | 1.50 |  |  |  |  |  |  | - |

*Note: Pearson correlations using listwise deletion; Grey shaded values mean non-significant coefficients; SES (Objective), was created by the sum of standardized responses of monthly family income participants reported and their education level; *** p<.001, **p<.01, *p<.05.*

# Supplementary analyses in Study 2

## Descriptive statistics and Correlations in Study 2

Table S2.
*Descriptive statistics and Correlations between variables in Study 2*

|  | *M* | SD | *1* | *2* | *3* | *4* | *5* | *6* | *7* | 8 |
| --- | --- | --- | --- | --- | --- | --- | --- | --- | --- | --- |
| *1. Status Consumption*  *Griskevicius, et al. Scale* | 3.85 | 1.35 | - | 0.260*** | 0.053 | 0.222*** | 0.246*** | 0.227*** | 0.061 | 0.185** |
| *2. Status Consumption*  *Based on O´Cass and McEwen* | 1.89 | 0.78 |  | - | 0.297*** | 0.505*** | 0.642*** | 0.618*** | -0.066 | 0.195*** |
| *3. Status Anxiety* | 3.16 | 0.81 |  |  | - | 0.554*** | 0.398*** | 0.270*** | -0.052 | 0.034 |
| *4. Status Seeking* | 3.94 | 1.20 |  |  |  | - | 0.494*** | 0.356*** | 0.026 | 0.119* |
| *5. Materialism* | 2.36 | 0.52 |  |  |  |  | - | 0.521*** | -0.147* | 0.173** |
| *6. Conspicuous Consumption* | 2.15 | 0.85 |  |  |  |  |  | - | -0.004 | 0.140* |
| *7. SES (Objective)* | 0.0060 | 1.43 |  |  |  |  |  |  | - | 0.133* |
| *8. S_SES (Subjective)* | 5.60 | 1.28 |  |  |  |  |  |  |  | - |

*Note: Pearson correlations using listwise deletion; Grey shaded values mean non-significant coefficients; SES (Objective), was created by the sum of standardized responses of monthly family income participants reported and their education level; *** p<.001, **p<.01, *p<.05.*
